# Effects of Probiotics, Prebiotics and Synbiotics Injected in Ovo on the Microstructure of the Breast Muscle in Different Chicken Genotypes

**DOI:** 10.3390/ani11102944

**Published:** 2021-10-12

**Authors:** Karolina Stasiak, Anna Slawinska, Joanna Bogucka

**Affiliations:** 1 Department of Animal Physiology and Physiotherapy, Faculty of Animal Breeding and Biology, Bydgoszcz University of Science and Technology, 85-084 Bydgoszcz, Poland; bogucka@pbs.edu.pl; 2 Department of Animal Biotechnology and Genetics, Faculty of Animal Breeding and Biology, Bydgoszcz University of Science and Technology, 85-084 Bydgoszcz, Poland; slawinska@pbs.edu.pl

**Keywords:** bioactive compounds, superficial pectoral muscle, muscle fibres, histopathology, necrotic fibres, fibre splitting

## Abstract

**Simple Summary:**

Probiotics, prebiotics and synbiotics are defined as biologically active food ingredients or food supplements. They promote higher growth performance but also have a positive effect on animal health by reducing the incidence of intestinal diseases and the risk of contamination of poultry products. These substances can be an alternative to recently banned antibiotics, used mainly to prevent infections, treat sick animals and promote growth. The present study compared the effects of different bioactive substances on the histological features of muscles from chickens representing two genotypes: Ross 308 broilers and GP native chickens. The results obtained clearly indicate that the microstructural features of pectoral muscles depend not only on the type of the injected bioactive substance but also on the genotype of the chickens.

**Abstract:**

The aim of the study was to analyse the effect of probiotics, prebiotics and synbiotics injected in ovo on day 12 of embryonic development on the microstructure of the superficial pectoral muscle (musculus pectoralis superficialis) from 42-day-old chickens of different genotypes: broilers (Ross 308) and general-purpose type (green-legged partridge (GP) chickens Zk-11, native chickens). Incubated eggs were divided into four groups (each genotype separately) depending on the substance injected in ovo: normal saline (C, control); *Lactococcus lactis* subsp. *cremoris* (PRO); galactooligosaccharides, GOS (PRE) or GOS + *L. lactis* (SYN). After hatching, chicks were placed in eight replicated pens (four pens/genotype group). There were eight birds per pen. In total, 64 birds were used in the experiment. Birds were slaughtered at the age of 42 days, and samples of superficial pectoral muscles were taken for analysis. The microstructure of the pectoral muscles was evaluated using the cryosectioning (frozen tissue sectioning) technique and staining with haematoxylin and eosin. Statistical analysis revealed that the in ovo injection of probiotics, prebiotics and synbiotics had no significant effect on the diameter of muscle fibres from chickens of the two genotypes. The number of fibres in the muscles from green-legged partridge chickens was about three-fold higher than the fibre density in the muscles from broiler chickens, with the fibre diameter being two-fold smaller. This fact may indicate a greater tenderness of meat from GP chickens compared to the meat from Ross 308 broilers. In the case of broilers, a prebiotic (GOS) was the most effective bioactive substance in reducing the number of histopathological changes. Considering muscles from GP chickens, the number of normal fibres was highest in birds treated with the probiotic. These findings indicate that the microstructural features of pectoral muscles depend not only on the type of the injected bioactive substance but also on the genotype of chickens.

## 1. Introduction

Probiotics, prebiotics and synbiotics are defined as biologically active food ingredients or food supplements that meet the basic human nutritional needs necessary for good health [1]. These substances can be an alternative to recently banned antibiotics, used mainly to prevent infections, treat sick animals and promote growth [2,3]. Intestinal diseases in birds are a serious problem faced by the poultry industry. Previous studies [4,5] have demonstrated that the use of bioactive substances reduces the incidence of these diseases and the subsequent contamination of poultry products. The action of different bioactive substances relies on different mechanisms.

Probiotics are products that contain viable microorganisms that modify, by im-plantation or colonisation, the microflora of the host [6]. These microorganisms act in the gastrointestinal tract by competing with ingested pathogens, enhancing the host’s physiology and promoting the digestion and absorption of nutrients, thereby producing a positive effect on the growth performance of animals [7,8]. Prebiotics, on the other hand, are sugars, mainly polysaccharides, which are not digested in the stomach and intestines of monogastric animals. They selectively stimulate the growth and/or activity of beneficial intestinal microflora and reduce the number of bacteria in the colon [9,10,11]. Prebiotics are nutrients used by bacteria that normally live in the digestive tract, which convert indigestible carbohydrates into a source of energy. Prebiotics also regulate fat metabolism and reduce the risk of hypoglycaemia, allergy and neoplastic diseases [11]. A synergistic combination of properly selected probiotic bacteria and a prebiotic, which is their source of energy, is defined as a synbiotic [12]. Both these compounds can act synergistically with each other (the prebiotic stimulates the growth of probiotic bacteria) or synergistically with the host (acting independently to stimulate the growth of the host’s microflora) (literature review in [13]). This action can be useful in improving gut health, which in turn can increase the feed conversion ratio and, therefore, promote the growth of birds. Improved intestinal health may also be reflected in the general metabolism [14,15]. Therefore, the use of synbiotics appears to be more effective than the administration of probiotics or prebiotics alone.

Gut microbiome modulation is influenced not only by the type of bioactive sub-stance and its dose but also by the time and method of its administration [16,17]. In routine practice, bioactive substances are dissolved in water and provided with feed to chicks immediately after hatching until the second week of age. During this time, the gastrointestinal tract is colonised for the first time by the microbiota and reaches functional maturity [18,19]. This period can be extended by the administration of prebiotics and probiotics (or their synergistic combination) using in ovo technology. Many studies have demonstrated that the optimal time to inject a small dose of a bioactive substance into the air chamber of a fertilised egg is on day 12 of incubation [15,20]. At this time, the chorioallantoic membrane is highly vascularised and a prebiotic can easily penetrate from the air chamber into the circulatory system and further to the developing intestine [17]. In turn, probiotics penetrate to the gastrointestinal tract in the early stages of hatching (i.e., day 19 of egg incubation) [15]. In this way, the microbiome of the developing embryo is exposed to a strong stimulating effect.

A number of studies confirmed the relationship between the gut microbiome and the metabolic pathways of substances absorbed in the intestine, increased muscle weight and differences in the parameters of meat quality [21,22,23]. However, data on the effect of in-ovo-delivered bioactive substances on meat quality and the microstructure of skeletal muscles in birds are inconsistent [15]. Some studies demonstrated a marked increase in the density of muscle fibres associated with the administration of prebiotics [21,24] or synbiotics [24,25], which was associated with higher meat tenderness. The positive effect of a prebiotic on fatty acid composition in chicken meat was reported by Angwech et al. [26], but a study by Tavaniello et al. [22] did not confirm this finding. On the other hand, Maiorano et al. [21] reported that the supplementation with a prebiotic had no significant effect on the content of intramuscular fat, while Dankowiakowska et al. [27] found a higher content of intramuscular fat, but in a study by Tavaniello et al. [22] this parameter was lower.

The limited number of studies investigating the effect of supplementation with a variety of bioactive compounds on the microstructure of skeletal muscles in birds prompted us to carry out this type of analysis. The aim of the study was to compare the microstructure of superficial pectoral muscles (musculus pectoralis superficialis) from 42-day-old chickens of different genotypes: broilers (Ross 308) and general-purpose type (green-legged partridge (GP) chickens, Zk-11 line; native chickens) and analyse the effects of a probiotic, prebiotic and synbiotic injected in ovo on day 12 of embryonic development on the features of these muscles in both genotypes.

## 2. Materials and Methods

### 2.1. Animals and in Ovo Injections

The experiment was carried out on chickens representing two genotypes: Ross 308 broilers (32 birds) and Zk-11 green-legged partridge (GP) chickens (32 birds). The experimental factor was a bioactive compound (probiotic—*Lactococcus lactis* subsp. *cremoris*, 10^5^ CFU/egg; prebiotic—GOS, galactooligosaccharides, 3.5 mg/egg, or synbiotic—GOS, 3.5 mg/egg + *L. lactis*, 10^5^ CFU/egg) injected in ovo into the air chamber of an egg on day 12 of embryogenesis. Control eggs were injected with sterile normal saline. All used bioactive compounds were prepared as aqueous solutions (in normal saline), and the injection volume was 0.2 mL. When selecting bioactive compounds, their immunostimulatory and adhesive properties were taken into account [13,28]. GOS is known under the trade name Bi^2^tos and was obtained from Clasado Biosciences Ltd. (Jersey, UK), whereas *Lactococcus lactis* subsp. *cremoris* IBB477 was derived from the collection of the Institute of Biochemistry and Biophysics Polish Academy of Sciences (Warsaw, Poland). The procedure for the in ovo delivery of bioactive compounds has been described in another paper [29]. All eggs were candled to eliminate unfertilised eggs and non-viable embryos. After hatching, chicks were placed in eight replicated pens (four pens: C—controls injected in ovo with normal saline, PRO—probiotic group injected with *Lactococcus lactis* subsp. *cremoris*, PRE—prebiotic group injected with GOS (galactooligosaccharides) and SYN—group injected with symbiotic (GOS + *Lactococcus lactis* subsp. *cremoris*)/genotype group), with a surface area of 3.75 m^2^ lined with litter, and with a stocking rate of 17.33 birds/m^2^. There were eight birds per pen. Bird management was consistent with guidelines provided by the Local Ethical Committee at UTP Bydgoszcz (no. 16/2014) and recommendations on animal welfare presented in Directive 2010/63/EU. The birds’ diet was consistent with recommendations for each lineage, age and genotype (Table 1). Broiler and GP chickens were slaughtered at the age of 42 days, and tissue samples were collected for analysis. In total, 64 birds were used in the experiment.

### 2.2. Breast Muscle Microstructure Evaluation 

The microstructure of the pectoral muscles was evaluated using the cryosectioning technique (frozen tissue sectioning). Samples of the superficial breast muscles were cut into 10 μm slices using a cryostat (Thermo Scientific, London, UK). Slices were transferred onto glass slides, placed in a freezer at −20 °C and kept until histochemical staining was performed. Breast muscle microstructure was evaluated for 64 slides stained in a single procedure.

### 2.3. Haematoxylin and Eosin Staining (H + E) 

Specimens were removed from the freezer, dried at room temperature, preserved in 4% formalin, placed in cuvettes with haematoxylin and next 0.1% eosin and then rinsed with distilled and running water. At the next stage, slides were placed in a series of cuvettes with 70%, 96% and 100% ethyl alcohol. Finally, the slides were treated with xylene and then sealed with coverslips using Leica CV Mount Medium Synthetic Adhesive. A Delta Optical Evolution 300 microscope with a ToupCam^TM^ camera (Warsaw, Poland) was used for the acquisition of histological images. Specimens of superficial pectoral muscles from chickens were evaluated under a microscope using MultiScanBase software v. 18.03 (Computer Scanning System II, Warsaw, Poland). The muscle fibres were measured for their diameter and density (number of fibres/1.5 mm^2^) and evaluated for histopathological changes. The following histopathological changes were quantified: muscle fibre atrophy, giant fibres, changes in the shape of fibres (triangular, trapezoidal, oblong), fibre necrosis with phagocytosis and fibre splitting.

### 2.4. Statistical Analysis

Acquired data were statistically analysed using STATISTICA 13.1 software (StatSoft Polska sp. z o.o., Krakow, Poland). Mean and standard deviations were calculated for each feature. Significance of differences between the experimental groups was estimated by one-way analysis of variance (ANOVA) and Tukey’s HSD test. Differences were considered significant at *p* < 0.05.

## 3. Results and Discussion

### 3.1. Effect of In-Ovo-Delivered Bioactive Compounds on Chicken Growth

To ensure the health of animals as well as the health of future consumers, a properly formulated animal diet seems to be of particular importance. An effective solution in feeding livestock is supplementation with probiotics, prebiotics or synbiotics. Currently, poultry production relies mainly on the hybrids of selected lines characterised by rapid growth. However, rapid weight gain in birds, with uneven development of the whole body, can often lead to many disorders (e.g., ascites, breast blisters or limb diseases), which have a negative impact on the health of birds and the quality of meat [30,31]. The birds used in our study differed in terms of origin, body size and growth rate [32]. Ross 308 broilers are fast-growing hybrids, while green-legged partridge chickens are a slow-growing old breed native to Poland. After six weeks of the experiment, the mean body weight of the GP chickens was approximately seven times lower than that of the broiler chickens (Table 2).

The analysis of data on the growth performance of birds used in the experiment revealed a significant effect of bioactive compounds on the body weight of Ross 308 broiler chickens (*p* < 0.05). At the end of the production period, the body weight was highest in birds from the group injected with a prebiotic (GOS), and it was on average 147.7 g higher compared to control birds. The lowest body weight was found in birds injected in ovo with the synbiotic (GOS + *L. lactis*). These broilers were on average 150.9 g lighter compared to control birds. Values obtained in the present experiment are consistent with findings reported by Dankowiakowska et al. [33], who investigated the effects of a prebiotic (Bi^2^tos) and a synbiotic (Bi^2^tos + *Lactococcus lactis* ssp. *cremoris*) on the microstructural features of superficial breast muscles in broiler chickens. According to Pruszyńska-Oszmalek et al. [14], the higher body weight of the birds may be caused by the increased activity of pancreatic enzymes and better absorption of nutrients in the gut [34]. Studies by other authors have revealed that the increase in the body weight of birds depends not only on the method of administration but also on the type of synbiotic used. No significant effect of a supplemental synbiotic (composed of prebiotic raffinose family oligosaccharides and probiotic *Lactobacillus lactis* or *Lactobacillus acidophilus* plus *Streptococcus faecium*) delivered in ovo on the growth of birds was also reported by Sławińska et al. [20] and Milczarek et al. [35] in their study on the use of synbiotics (including a carbohydrate preparation derived from yeast *Saccharomyces cerevisiae* enriched with *Bacillus subtilis* bacteria). On the other hand, a study by Abdel-Hafeez et al. [36] demonstrated a significant increase in the body weight of chickens (Arbor Acres) fed a diet supplemented with a synbiotic after a short time, in the second week of production. Meanwhile, in our study, the in ovo injection of the synbiotic (GOS + *L. lactis*) was associated with the lowest body weight gains observed early, in the third week of production. In contrast to the performance of broilers, none of the used bioactive compounds had a significant effect on the body weight of GP chickens. Differences in the body weight of 42-day-old GP chickens (419.2 g for the SYN and 465.3 g for the PRE) were not statistically significant. Significant differences between groups that were injected in ovo with different bioactive compounds in the body weight of GP chickens were only observed between weeks two and four of production. During this period, chickens that were injected with the synbiotic were characterised by the smallest body weight gains compared to other treatment groups.

### 3.2. Breast Muscle Microstructure in Broilers and Native Chickens

Muscle mass is determined by the total number of muscle fibres, their size and type [37]. The structure of skeletal muscle varies considerably between animal species and breeds. According to Karlsson et al. [38], the histological features of skeletal muscles are influenced by sex, age, prenatal and postnatal nutrition, production system (air humidity, temperature, access to outdoor pens) and selection strategy (birds for egg or meat production). Microstructural features of pectoral muscles from the examined chickens are presented in Table 3. Despite the clear trend towards a decreasing diameter of the muscle fibres, statistical analysis did not reveal a significant influence of bioactive substances injected in ovo on this parameter. Similar observations have been made in other studies investigating the influence of various bioactive substances on the microstructure of chicken breast muscles [24,33,39]. According to Bogucka et al. [25], the trend towards reduced muscle fibre diameter with a simultaneous increase in their density has a positive effect on meat quality. The number of fibres in the muscles from green-legged partridge chickens was about three-fold higher than the fibre density in the muscles of broiler chickens, with a two-fold smaller fibre diameter. This fact may indicate a greater tenderness of meat from GP chickens compared to the meat from Ross 308 broilers. 

### 3.3. Effect of in Ovo Injection of Bioactive Compounds on Breast Muscle Microstructure

Bioactive substances used in the present study (for broilers: probiotic and prebiotic; for green-legged partridge chickens: probiotic, prebiotic and synbiotic) had a positive effect on the percentage of normal muscle fibres. In Ross 308 chickens, the percentage of normal fibres was highest in the group injected with the prebiotic (95.02%) and lowest in the group injected with the synbiotic (92.60%) (*p* < 0.05). These results are consistent with findings by Cianciullo [40]. In the examined muscles from Ross 308 chickens, the percentage of normal fibres reported by Cianciullo was 93.63% for the prebiotic group (RFOs) and 94.78% for the synbiotic group (RFOs + *Lactococcus lactis* ssp. *cremoris*). Compared to broilers, the breast muscles from GP chickens were characterised by a much greater number of normal fibres (96.32% to 98.21%). Considering this feature, the muscles of GP chickens resemble those of turkeys. According to Górska and Wojtysiak [41], breast muscles from turkeys contained 97.32% of normal fibres. A greater percentage of normal muscle fibres indicates a lower number of histopathological changes. A study by Elminowska-Wenda et al. [42] revealed that histopathological changes are most extensive in fast growing birds characterised by high meatiness. The selection of birds representing different genotypes is apparently a perfect confirmation of this claim.

### 3.4. Histopathological Changes in the Breast Muscle in Broilers and Native Chickens Injected in Ovo with Bioactive Compounds

**Figure 1:**
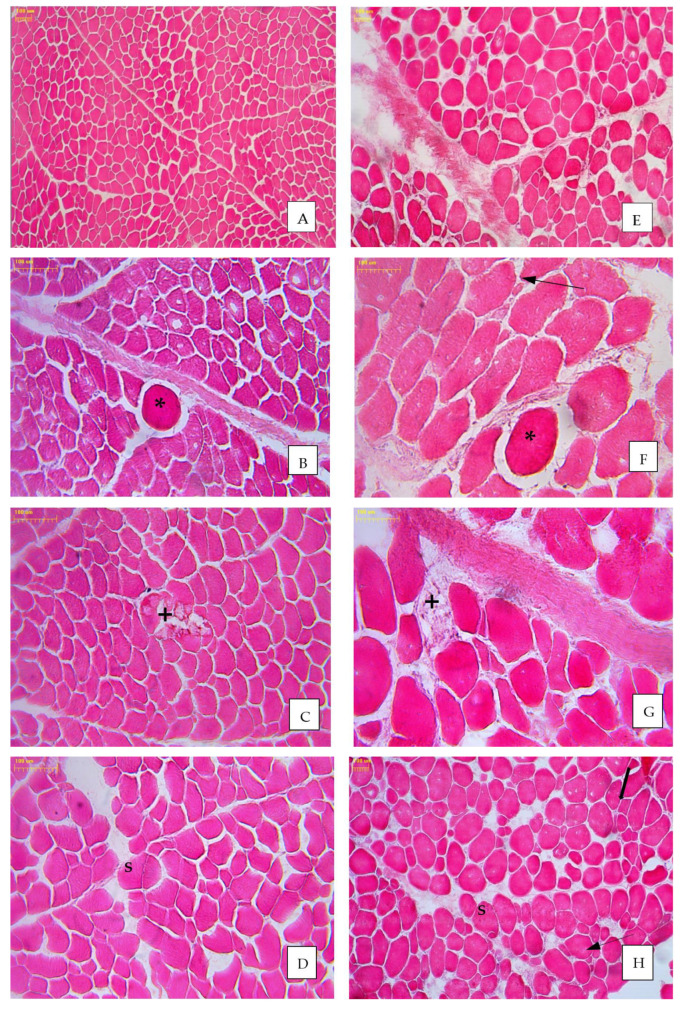
Microstructure of pectoral muscle of green-legged partridge (GP) chickens (**A**–**D**) and Ross 308 broilers chicken (**E**–**H**); HE stain; * giant fibres; ^+^—necrotic fibres; S—fibre splitting; change shape of fibre (thick arrow); atrophic fibres (thin arrows); magnification ×100 (**A**,**E**,**H**); magnification ×200 (**B**–**D**,**F**,**G**).

Muscle tissue is very sensitive to harmful factors. Histopathological changes may result from hypoxia, inflammation or electrolyte disorders, as well as excess of calcium in cells or hypertrophy of connective tissue [43,44]. The above-mentioned factors not only change the appearance of meat but also its chemical composition [45]. A reduced proportion of normal muscle fibres was associated with the presence of changes such as muscle fibre atrophy, giant fibres, changes in the shape of fibres, fibre necrosis and fibre splitting (Figure 1). Considering all these anomalies, significant differences between the studied groups of broilers were observed only for changes in the shape of fibres. The percentage of fibres with abnormal shape detected in the cross-section was lowest in the prebiotic group (1.68%), and it was significantly different from the synbiotic group (3.03%) (*p* < 0.05). Considering this feature, a positive effect of bioactive substances injected on day 12 of embryonic development was observed in GP chickens. The percentage of fibres with abnormal shape in the control group of GP chickens (1.3%) was higher than in other treatment groups: 0.48% for the probiotic; 0.73% for the prebiotic and 0.91% for the synbiotic.

#### 3.4.1. Fibre Atrophy

Another histopathological change identified in approximately 2% of analysed breast muscles from broiler chickens and in approximately 1% of breast muscles from GP chickens was fibre atrophy. This anomaly is mainly caused by an insufficient supply of nutrients or the predominance of catabolism due to the overall reduction in the metabolic rate [46]. Supplementation with bioactive substances did not significantly reduce the percentage of atrophic fibres in breast muscles from broiler chickens. On the other hand, in GP chickens from all treatment groups, the percentage of atrophic fibres was reduced compared to that of controls, and the greatest reduction was found in the probiotic group (0.42%) and the synbiotic group (0.47%). 

#### 3.4.2. Giant Fibres

The presence of giant fibres (GFs) is linked by many researchers with the intensive genetic selection of chicken [43,47]. As a result, rapid muscle growth and development may lead to the pathological hypertrophy of muscle fibres [48]. Thus far, however, no negative effect of these fibres on the quality of chicken meat has been reported. Giant fibres were identified in muscles from all groups of chickens. The percentage of GFs was clearly influenced by the genotype of birds (it was two-fold lower in the muscles from Zk-11 chickens compared to that in broiler chickens). Our study did not reveal any significant effect of in ovo delivery of bioactive substances on the presence of GFs in breast muscles from broiler chickens. A similar conclusion was reached in a study by Bogucka et al. [25], who reported an extremely low percentage of giant fibres (0.04%) in analysed muscles from Ross 308 chickens. In contrast, the percentage of GFs clearly reduced in GP chickens assigned to PRO or SYN treatments (from 0.43% to 0.14%; *p* < 0.05).

#### 3.4.3. Necrotic Fibres

Necrotic fibres (responsible for meat spoilage) are formed in muscles in response to hypoxia, which might be caused by the low proliferation of capillaries or occlusion of the arteries [43]. On the other hand, Sandercock et al. concluded that the formation of necrotic fibres is associated with an excess of intracellular calcium in fibres [45]. In all treatment groups (both Ross 308 and GP chickens), the use of bioactive compounds was associated with a reduced percentage of necrotic fibres in muscles. However, significant differences were only found for green-legged partridge chickens. Similar observations were made in a study by Bogucka et al. [25], who investigated the effect of a synbiotic on the quality of meat from Ross 308 chickens. The difference between the number of necrotic fibres in the control group and in birds treated with the synbiotic (prebiotic RFO + probiotic Lavipan®) was not statistically significant. On the other hand, in GP chickens, a positive effect was achieved in the group of birds injected with the synbiotic (only 0.13% of necrotic fibres), while the prebiotic was the least effective bioactive substance in reducing this histopathological change (the number of necrotic fibres decreased on average by 0.18%).

#### 3.4.4. Fibre Splitting

Fibre splitting was another degenerative change observed in muscle fibres from all the examined birds. This term refers to the longitudinal splitting of muscle fibres and the formation of several thinner fibres. According to MacRae et al. [49], splitting is an adaptive response to metabolic stress associated, for example, with the functioning of some hypertrophic (larger) fibres. The present study demonstrated that the prebiotic had the strongest effect on the percentage of abnormal split fibres. Both in broiler chickens and GP chickens, the in ovo injection of the prebiotic (GOS) was associated with the greatest, yet not statistically significant, reduction in the number of split fibres. On the other hand, unlike in the study by Bogucka et al. [25], the use of a synbiotic (GOS + *L. lactis*) was associated with an increased number of split fibres not only in broiler chickens but also in GP chickens. However, this increase was not statistically significant. The conducted experiment confirmed a significant effect of the genotype of chickens on the number of split fibres. The number of split fibres in the muscles from GP chickens was two-fold lower compared to that in breast muscles from broiler chickens.

## 4. Conclusions

There is a limited number of published studies on the effect of probiotics, prebiotics and synbiotics injected in ovo on the microstructural features of the superficial pectoral muscle (musculus pectoralis superficialis) in chickens. The present study compared the effects of different bioactive substances on the histological features of muscles from chickens representing two genotypes: Ross 308 broilers and GP native chickens. In broilers, the use of a prebiotic (GOS) was most effective in reducing the number of histopathological changes. In GP chickens, the number of normal muscle fibres was highest in the probiotic group of birds that were injected in ovo with *L. lactis* subsp. *cremoris*. These findings clearly indicate that the microstructural features of pectoral muscles depend not only on the type of the injected bioactive substance but also on the genotype of chickens.

## Figures and Tables

**Table 1:** Chemical composition of commercial feeds used for chicken broilers and green-legged partridge chickens.

Items	Broilers Ross 308				Green-Legged Partridge	
	Starter (Days 1–10)	Grower I (Days 11–21)	Grower II (Days 22–33)	Finisher (Days 34–42)	Starter (Days 1–28)	Grower (Days 19–42)
ME_N_ (MJ/kg)	12.50	12.95	13.35	13.41	11.9	11.7
Crude protein (g/kg)	220	200	190	184	200	185
Crude fibre (g/kg)	28.00	30.00	31.00	32.00	34.00	35.00
Lysine (g/kg)	13.8	12.5	11.3	10.5	11.0	10.0
Methionine + cystine (g/kg)	10.3	9.5	8.8	8.2	8.2	7.2
Threonine (g/kg)	9.2	8.3	7.6	7.2	7.6	7.0
Tryptophan (g/kg)	2.2	2.0	1.9	1.9	2.1	2.0

**Table 2:** Body weight of broiler chickens and green-legged partridge chickens during the production period (means ± SD).

Breed/Group		1 Week	2 Week	3 Week	4 Week	5 Week	6 Week
Broilers Ross 308	C	194.3 ^b^ ± 16.3	522.1 ^b^ ± 56.5	990.5 ^bc^ ± 93.1	1723.8 ^bc^ ± 120.3	2565.9 ^bc^ ± 165.8	3129.4 ^b^ ± 219.6
	PRO	204.9 ^a^ ± 17.7	556.2 ^a^ ± 52.2	1049.7 ^a^ ± 95.2	1799.6 ^a^ ± 109.5	2702.6 ^a^ ± 163.8	3229.7 ^ab^ ± 320.7
	PRE	195.6 ^b^ ± 23.0	544.1 ^ab^ ± 73.4	1019.0 ^ab^ ± 135.9	1782.9 ^ab^ ± 148.1	2621.4 ^ab^ ± 284.1	3277.1 ^a^ ± 325.2
	SYN	198.7 ^ab^ ± 18.9	529.0 ^b^ ± 56.5	972.7 ^c^ ± 102.5	1712.4 ^c^ ± 128.1	2492.6 ^c^ ± 178.2	2978.5 ^c^ ± 243.7
Green-legged Partridge	C	83.2 ^ab^ ± 8.8	150.1 ^a^ ± 21.0	230.4 ^a^ ± 36.9	325.9 ^a^ ± 53.8	419.1 ± 72.0	446.2 ± 77.7
	PRO	83.4 ^ab^ ± 8.8	154.7 ^a^ ± 14.4	239.5 ^a^ ± 19.6	331.3 ^a^ ± 29.3	437.4 ± 39.8	448.8 ± 47.4
	PRE	85.2 ^a^ ± 8.0	150.2 ^a^ ± 24.6	238.5 ^a^ ± 31.2	314.0 ^ab^ ± 43.6	418.3 ± 79.2	465.3 ± 57.1
	SYN	81.6 ^b^ ± 8.5	142.2 ^b^ ± 17.3	199.8 ^b^ ± 36.8	299.4 ^b^ ± 52.3	417.5 ± 54.1	419.2 ± 56.2

^a,b,c^ Mean values in the columns within one breed marked with different letters differ significantly (*p* < 0.05). C—controls injected in ovo with normal saline. PRO—probiotic group injected with *Lactococcus lactis* subsp. *cremoris*; PRE—prebiotic group injected with GOS (galactooligosaccharides); SYN—group injected with symbiotic (GOS + *Lactococcus lactis* subsp. *cremoris*).

**Table 3:** Microstructural features and the percentage (%) of normal and pathological fibres in the superficial breast muscles from 42-day-old Ross 308 broiler chickens and green-legged partridge (GP) chickens injected in ovo with bioactive substances on day 12 of embryonic development (means ± SD).

Breed/Group		Traits							
		Fibre Dimeter (μm)	Muscle Fibre Dimeter (Fibre Number/1.5 mm^2^)	Normal Fibres (%)	Fibre Atrophy (%)	Giant Fibres (%)	Changes in the Shape of Fibres (%)	Necrotic Fibres (%)	Fibre Splitting (%)
Broilers Ross 308	C	57.63 ^x^ ± 5.38	239.13 ^y^ ± 42.40	93.02 ^aby^ ± 2.22	1.66 ^ax^ ± 0.55	0.70 ^ax^ ± 0.42	2.52 ^abx^ ± 0.78	1.22 ^ax^ ± 0.60	1.08 ^ax^ ± 0.47
	PRO	51.52 ^x^ ± 6.76	244.75 ^y^ ± 50.46	93.32 ^aby^ ± 1.71	1.63 ^ax^ ± 0.63	0.88 ^ax^ ± 0.34	2.13 ^abx^ ± 0.48	0.97 ^ax^ ± 0.65	1.12 ^ax^ ± 0.60
	PRE	56.21 ^x^ ± 6.10	243.50 ^y^ ± 55.94	95.02 ^ay^ ± 1.30	1.08 ^ax^ ± 0.56	0.56 ^ax^ ± 0.25	1.68 ^bx^ ± 0.75	0.89 ^ax^ ± 0.53	0.77 ^ax^ ± 0.28
	SYN	53.82 ^x^ ± 5.68	244.86 ^y^ ± 43.73	92.60 ^by^ ± 1.03	1.74 ^ax^ ± 0.28	0.56 ^ax^ ± 0.37	3.03 ^ax^ ± 0.81	0.94 ^ax^ ± 0.46	1.26 ^ax^ ± 0.46
Green-legged Partridge	C	25.65 ^y^ ± 1.77	871.38 ^x^ ± 108.50	96.32 ^ax^ ± 0.54	1.03 ^ay^ ± 0.10	0.43 ^ax^ ± 0.20	1.30 ^ay^ ± 0.37	0.47 ^ay^ ± 0.22	0.44 ^ay^ ± 0.19
	PRO	23.09 ^y^ ± 2.72	897.38 ^x^ ± 95.48	98.21 ^ax^ ± 0.53	0.42 ^by^ ± 0.14	0.14 ^cy^ ± 0.17	0.48 ^by^ ± 0.30	0.23 ^by^ ± 0.11	0.51^ay^ ± 0.29
	PRE	24.36 ^y^ ± 2.64	883.00 ^x^ ± 158.66	97.68 ^bx^ ± 0.86	0.82 ^cx^ ± 0.19	0.20 ^acy^ ± 0.12	0.73 ^bcy^ ± 0.36	0.29 ^aby^ ± 0.19	0.41^ay^ ± 0.15
	SYN	24.19 ^y^ ± 2.29	878.00 ^x^ ± 113.37	97.73 ^ax^ ± 0.52	0.47 ^bdy^ ± 0.13	0.14 ^cdy^ ± 0.18	0.91 ^acy^ ± 0.20	0.13 ^by^ ± 0.07	0.62 ^ay^ ± 0.33

^a,b,c,d^ Statistically significant differences between treatment groups (C, PRO, PRE and SYN) within the same genetic group of chickens at *p* < 0.05; ^x,y^ statistically significant differences between treatment groups (C, PRO, PRE and SYN) and between genetic groups of chickens at *p* < 0.05; C—controls injected in ovo with normal saline. PRO—probiotic group injected with *Lactococcus lactis* subsp. *cremoris*; PRE—prebiotic group injected with GOS (galactooligosaccharides); SYN—group injected with symbiotic (GOS + *Lactococcus lactis* subsp. *cremoris*).

## Data Availability

The data presented in this study are available on request from the corresponding author.

## References

[1] Guaadaoui A., Benaicha S., Elmajdoub N., Bellaoui M., Hamal A. (2014). What is a bioactive compound? A combined definition for a preliminary consensus. Int. J. Food Nutr. Sci..

[2] Hume H.E. (2011). Historic perspective: Prebiotics, probiotics and other alternatives to antibiotics. Poult Sci..

[3] Young I., Rajic A., Wilhelm B.J., Waddell L., Parkew S., Mcewen S.A. (2009). Comparison of the prevalence of bacterial enteropathogens, potentially zoonotic bacteria and bacterial resistance to antimicrobials in organic and conventional poultry, swine and beef production: A systematic review and meta-analysis. Epidemiol. Infect..

[4] Fioramonti J., Theodorou V., Bueno L. (2003). Probiotics: What are they? What are their effects on gut physiology?. Best Pract. Res. Clin. Gastroenterol..

[5] Siragusa G.R., Ricke S.C., Ricke S.C., van Loo E.J., Johnson M.G., O’Bryan C.A. (2012). Probiotics as pathogen control agents for organic meat production. Organic Meat Production and Processing.

[6] Havenaar R., Huis in’t Veld J.H.J., Wood B.J.B. (1992). Probiotics: A general view. The Lactic Acid Bacteria.

[7] Bozkrut M., Küçükyilmaz V.A., Çabuk M., Çatli A.U. (2011). Performance of layer or broiler breeder hens varies in response to different probiotic preparations. Ital. J. Anim. Sci..

[8] Younis T.M., El-Shafei A.A., Al-Gamal M.A., El-Sayed A.L. (2013). Effects of commercial probiotics on productive and physiological performance of broiler chickens. J. Appl. Sci. Res..

[9] Gibson G.R., Roberfroid M.B. (1995). Dietary modulation of the human colonic microbiota: Introducing the concept of prebiotics. J. Nutr..

[10] Gibson G.R. (1999). Dietary modulation of the human gut microflora using the prebiotics oligofructose and inulin. J. Nutr..

[11] Szymańska-Czerwińska M., Bednarek D. (2007). Beta-glucans as an alternative to antibiotic growth promoters. Życie Wet..

[12] Collins M.D., Gibson G.R. (1999). Probiotics, prebiotics, and synbiotics: Approaches for modulating the microbial ecology of the gut. Am. J. Clin. Nutr..

[13] Dunislawska A., Slawinska A., Stadnicka K., Bednarczyk M., Gulewicz P., Józefiak D., Siwek M. (2017). Synbiotics for broiler chickens–in vitro design and evaluation of the influence on host and selected microbiota populations following in ovo delivery. PLoS ONE.

[14] Pruszyńska-Oszmalek E., Kolodziejski P.A., Stadnicka K., Sassek M., Chalupka D., Kuston B., Nogowski L., Mackowiak P., Maiorano G., Jankowski J. (2015). In ovo injection of prebiotics and synbiotics affects the digestive potency of the pancreas in growing chickens. Poult. Sci..

[15] Siwek M., Slawinska A., Stadnicka K., Bogucka J., Dunislawska A., Bednarczyk M. (2018). Prebiotics and synbiotics—In ovo delivery for improved lifespan condition in chicken. BMC Vet. Res..

[16] De Vrese M., Schrezenmeir J. (2008). Probiotics, prebiotics, and synbiotics. Adv. Biochem. Eng. Biotechnol..

[17] Tavaniello S., Maiorano G., Stadnicka K., Mucci R., Bogucka J., Bednarczyk M. (2018). Prebiotics offered to broiler chicken exert positive effect on meat quality traits irrespective of delivery route. Poult. Sci..

[18] Sansonetti P.J., Di Santo J.P. (2007). Debugging how bacteria manipulate the immune response. Immunity.

[19] Bar-Shira E., Sklan D., Friedman A. (2003). Establishment of immune competence in the avian GALT during the immediate post-hatch period. Dev. Comp. Immunol..

[20] Sławińska A., Siwek M.Z., Bednarczyk M.F. (2014). Effects of synbiotics injected in ovo on regulation of immune-related gene expression in adult chickens. Am. J. Vet. Res..

[21] Maiorano G., Stadnicka K., Tavaniello S., Abiuso C., Bogucka J., Bednarczyk M. (2017). In ovo validation model to assess the efficacy of commercial prebiotics on broiler performance and oxidative stability of meat. Poult. Sci..

[22] Tavaniello S., Mucci R., Stadnicka K., Acaye O., Bednarczyk M., Maiorano G. (2019). Effect of in ovo administration of different synbiotics on carcass and meat quality traits in broiler chickens. Poult. Sci..

[23] Park J.H., Kim I.H. (2014). Supplemental effect of probiotic Bacillus subtilis B2A on productivity, organ weight, intestinal Salmonella microflora, and breast meat quality of growing broiler chicks. Poult. Sci..

[24] Maiorano G., Sobolewska A., Cianciullo D., Walasik K., Elminowska-Wenda G., Sławińska A., Tavaniello S., Żylińska J., Bardowski J., Bednarczyk M. (2012). Influence of in ovo prebiotic and synbiotics administration on meat quality of broiler chickens. Poult. Sci..

[25] Bogucka J., Ribeiro D.M., da Costa R.P., Bednarczyk M. (2018). Effect of synbiotic dietary supplementation on histological and histopathological parameters of *Pectoralis Major* muscle of broiler chickens. Czech J. Anim. Sci..

[26] Angwech H., Tavaniello S., Ongwech A., Kaaya A.N., Maiorano G. (2019). Efficacy of in ovo delivered prebiotics on growth performance, meat quality and gut health of kuroiler chickens in the face of a natural coccidiosis challenge. Animals.

[27] Dankowiakowska A., Bogucka J., Szczerba A., Sobolewska A., Kozlowska I., Maiorano G., Tavaniello S., Bednarczyk M. (2014). Assessment of intramuscular fat content and cholesterol content in M. pectoralis superficialis of chicken injected in ovo with bioactive substances. Book of Abstracts XXVI PO WPSA 8-10.09.

[28] Bednarczyk M., Stadnicka K., Kozłowska I., Abiuso C., Tavaniello S., Dankowiakowska A., Sławińska A., Maiorano G. (2016). Influence of different prebiotics and mode of their administration on broiler chicken performance. Animal.

[29] Slawinska A., Dunislawska A., Plowiec A., Radomska M., Lachmanska J., Siwek M., Tavaniello S., Maiorano G. (2019). Modulation of microbial communities and mucosal gene expression in chicken intestines after galactooligosaccharides delivery in ovo. PLoS ONE.

[30] Reiter K., Bessei W. (1998). Effect of locomotor activity on bone development and leg disorders in broiler. Arch. Geflügelk..

[31] Bosco A.D., Mugnai C., Amato M.G., Piottoli L., Cartoni A., Castellini C. (2014). Effect of slaughtering age in different commercial chicken genotypes reared according to the organic system: 1. Welfare, carcass and meat traits. Ital. J. Anim. Sci..

[32] Siwek M., Wragg D., Sławińska A., Malek M., Hanotte O., Mwacharo J.M. (2013). Insights into the genetic history of Green-legged Partridgelike fowl: mtDNA and genome-wide SNP analysis. Anim. Genet..

[33] Dankowiakowska A., Bogucka J., Sobolewska A., Tavaniello S., Maiorano G., Bednarczyk M. (2019). Effects of in ovo injection of prebiotics and synbiotics on the productive performance and microstructural features of the superficial pectoral muscle in broiler chickens. Poult. Sci..

[34] Sobolewska A., Bogucka J., Dankowiakowska A., Elminowska-Wenda G., Stadnicka K., Bednarczyk M. (2017). The impact of synbiotic administration through in ovo technology on the microstructure of a broiler chicken small intestine tissue on the 1st and 42nd day of rearing. J. Anim. Sci. Biotechnol..

[35] Milczarek A., Osek M., Olkowski B., Klocek B. (2012). Effect of probiotic, prebiotic and synbiotic on weight and pH of gastrointestinal tract in broiler chickens fed diets based on different cereals. Rocz. Nauk. Zootech..

[36] Abdel-Hafeez H.M., Saleh E.S.E., Tawfeek S.S., Youssef I.M.L., Abdel-Daim A.S.A. (2017). Effects of probiotic, prebiotic, and synbiotic with and without feed restriction on performance, hematological indices and carcass characteristics of broiler chickens. Asian-Australas J. Anim. Sci..

[37] Rehfeldt C., Stickland N.C., Fiedler I., Wegner J. (1999). Environmental and genetic factors as sources of variation on skeletal muscle fiber number. Basic Appl. Myol..

[38] Karlsson A.H., Enfalt A., Essen-Gustavsson B., Lundstrom K., Rydhmer I., Stern S. (1993). Muscle histochemical and biochemical properties in relation to meat quality during selection for increased lean tissue growth rate in pigs. J. Anim. Sci..

[39] Zhou X., Jin E., Shenghe L., Wang C., Qiao E., Guozhong W. (2015). Effects of dietary supplementation of probiotics (*Bacillus subtilis,*
*Bacillus licheniformis* and *Bacillus nato*) on broiler muscle development and meat quality. Turk. J. Vet. Anim. Sci..

[40] Cianciullo D. (2012). Effect of Prebiotic and Synbiotic Injected in ovo on Performance, Meat Quality and Histopathological Changes in Muscle of Broiler Chickens. Ph.D. Thesis.

[41] Górska M., Wojtysiak D. (2017). Pathological Changes in the Microstructure of Pale, Soft, Exudative (PSE) and Normal Turkey Breast Muscle. Folia Biol..

[42] Elminowska-Wenda G., Rosinski A., Baeza E. (2004). Histopathological changes in Pectoralis muscle of Landaise geese kept in different feeding system. Zesz. Nauk. AR Wroclaw. Zootech. L.

[43] Dransfield E., Sośnicki A.A. (1999). Relationship between muscle growth and poultry meat quality. Poult. Sci..

[44] Sandercock D.A., Barker Z.E., Mitchel M.A., Hocking P.M. (2009). Changes in muscle cell cation regulation and meat quality traits are associated with genetic selection for high body weight and meat yield in broiler chickens. Genet. Sel. Evol..

[45] Mazzoni M., Petracci M., Meluzzi A., Cavani C., Clavenzani P., Sirri F. (2015). Relationship between pectoralis major muscle histology and quality traits of chicken meat. Poult. Sci..

[46] Borisov A.B., Dedkov E.I., Carlson B.M. (2001). Interrelations of myogenic response, progressive atrophy of muscle fibers and cell death in denervated skeletal muscle. Anat. Rec..

[47] Remignon H., Zanusso J., Gaelle A., Babile R. (2000). Occurrence of giant myofibres according to muscle type, pre- or postrigor state and genetic background in turkeys. Meat Sci..

[48] Marcu A., Vacaru-Opris I., Dumitrescu G., Marcu A., Ciochina L.P., Nicula M., Dronca M., Kelciov B. (2013). Effect of diets with different energy and protein levels on breast muscle characteristics of broiler chickens. J. Anim. Sci. Biotechnol..

[49] MacRae V.E., Mahon M., Gilpin S., Sandercock D.A., Mitchell M.A. (2006). Skeletal muscle fiber growth and growth associated myopathy in the domestic chicken (*Gallus domesticus*). Br. Poult. Sci..

